# BMSC-EV-derived lncRNA NORAD Facilitates Migration, Invasion, and Angiogenesis in Osteosarcoma Cells by Regulating CREBBP *via* Delivery of miR-877-3p

**DOI:** 10.1155/2022/8825784

**Published:** 2022-03-01

**Authors:** Dapeng Feng, Zhengwei Li, Liang Yang, Haidong Liang, Hongtao He, Lin Liu, Wei Zhang

**Affiliations:** ^1^Department of Spine Surgery, The Second Hospital of Dalian Medical University, No. 467, Zhongshan Road, Shahekou District, Dalian, 116023 Liaoning, China; ^2^Department of Joint Surgery, The Second Hospital of Dalian Medical University, No. 467, Zhongshan Road, Shahekou District, Dalian, 116023 Liaoning, China; ^3^Department of Hands and Feet Microsurgery, The Second Hospital of Dalian Medical University, No. 467, Zhongshan Road, Shahekou District, Dalian, 116023 Liaoning, China; ^4^Department of Traumatology and Orthopedics, The Second Hospital of Dalian Medical University, No. 467, Zhongshan Road, Shahekou District, Dalian, 116023 Liaoning, China; ^5^Department of Neurology, The First Affiliated Hospital of Dalian Medical University, No. 222, Zhongshan Road, Xigang District, Dalian, 116011 Liaoning, China

## Abstract

Bone marrow mesenchymal stem cells (BMSCs) can boost osteosarcoma (OS) cell proliferation and invasion, yet the function of extracellular vesicles (EVs) derived from BMSCs on OS is scarcely known. This study is aimed at examining the role of BMSC-EVs in OS cells. BMSCs and BMSC-EVs were isolated and identified. The effect of EVs and EVs-si-NORAD on OS cell proliferation, invasion, migration, and angiogenesis was determined. Expressions of NORAD, miR-877-3p, and CREBBP were detected. The binding relationship among NORAD, miR-877-3p, and CREBBP was verified. The miR-877-3p inhibitor or pc-CREBBP was delivered into OS cells treated with EVs-si-NORAD for *in vitro* analysis. The nude mouse model of the subcutaneous tumor xenograft was established for *in vivo* analysis. BMSC-EVs promoted OS cell proliferation, invasion, migration, and angiogenesis. BMSC-EVs carried NORAD into OS cells and upregulated CREBBP by sponging miR-877-3p. miR-877-3p downregulation or CREBBP overexpression partly inverted the inhibitory effect of EVs by silencing NORAD on OS cell proliferation, invasion, migration, and angiogenesis. *In vivo* experiments validated that BMSC-EV-derived NORAD facilitated tumor growth by upregulating CREBBP *via* miR-877-3p. To conclude, BMSC-EV-derived NORAD facilitated OS cell proliferation, invasion, migration, and angiogenesis by modulating CREBBP *via* miR-877-3p, which may offer new insights into OS treatment.

## 1. Introduction

Osteosarcoma (OS) is one of the malignant tumors of bone with complex etiology and is common among adolescents and teenagers during fast growth in the metaphyseal of long bones, which accounts for 60% clinical incidence in all malignant tumors of bone in teenagers [1, 2]. Due to the features of high malignancy and rapid growth, patients are generally diagnosed with an advanced OS that is accompanied by metastasis and low survival rates [3]. Tumor angiogenesis plays a significant part in the tumorigenesis and metastasis of OS [4]. Thereafter, it is imperative to study the pathogenesis of OS and explore the potential biomarkers and therapeutic targets for OS treatment.

Bone marrow mesenchymal stem cells (BMSCs) as definitive components of the microenvironment are implicated in tumor cell proliferative and migratory abilities and drug tolerance in malignant tumors including OS [5]. An existing study elicited that the recruitment of BMSCs by OS sites induces an ameboid transition of cellular cytokines, thus leading to OS malignant transformation [6]. BMSCs can secrete extracellular vesicles (EVs) to affect OS [7]. EVs are cell-derived particles in the body fluid including apoptotic bodies, exosomes, and microbubbles, which have been ascertained to mediate OS progression [8, 9]. However, the definitive molecular mechanism remains unclear.

EVs can carry cargoes including long noncoding RNAs (lncRNAs) to participate in cell growth in cancers [10]. lncRNAs are a set of RNAs with a length of over 200 nucleotides extensively involved in a series of cellular processes, such as proliferation, migration, and invasion, and other biological processes such as genetic modulation and immune monitoring [11, 12]. Aberrant expressions of lncRNAs are commonly documented in all types of human cancers including OS, which serves as a prognostic biomarker for predicting clinical survival rates of cancer patients and exerts functional regulatory effects on the occurrence and development of tumor cells [13, 14]. The differential expressions of noncoding RNA activated by DNA damage (NORAD), a lncRNA composed of an exon located on Chr20q11.23, in renal cancer [15], gastric cancer [16], lung cancer [17], and OS [18] have been well-acknowledged, and the alteration of NORAD can manipulate cancer progression. However, whether NORAD mediates the regulation of BMSC-EVs on OS requires further investigation.

Similar to lncRNAs, microRNAs (miRNAs) are also non-protein-encoded RNAs with a length of about 18-22 nucleotides that can not only interact with lncRNAs but also regulate the expression of downstream mRNAs [19]. Moreover, miRNAs are regarded as important diagnostic and prognostic factors of OS and are widely applied in targeted therapies [20]. Based on the microarray analysis of circRNA expression, miR-877-3p has a great potential to act as the target miRNA of circRNA in OS progression [21]. Equally, the involvement of miR-877-3p in OS regulation as the downstream target miRNA of NORAD has not been reported yet. In addition, the analysis of 13 invasive OS tissues by array-based comparative genomic hybridization revealed a high amplification of histone acetyltransferases cAMP response element binding-binding protein (CREBBP) in OS, indicative of the involvement of CREBBP in OS development [22]. Nevertheless, the action of CREBBP as the target of miR-877-3p in OS invasion and angiogenesis should be further clarified. This study explored the mechanism of BMSC-EV-derived NORAD in the OS pathological process with the expectation to offer novel targets for OS diagnosis and management.

## 2. Materials and Methods

### 2.1. Ethics Statement

All animal experimental procedures were ratified by the Animal Ethics Committee of Dalian Medical University. The study was experimented in strict compliance with the Guidelines for the Care and Use of Laboratory Animals published in 2011. The number of animals and their suffering were minimized with best efforts.

### 2.2. Animals

Balb/c nude mice (4 weeks old) from Vital River Laboratory Animal Technology (SCXK (Beijing) 2016-0006, Beijing, China) were adaptively fed for 1 week before the experiments at 24-26°C and 50-60% humidity with free access to water and food.

### 2.3. Isolation and Identification of BMSCs

Balb/c mice were intraperitoneally administered with 800 mg/kg pentobarbital sodium for euthanasia, followed by the removal of the femur and tibia under sterile conditions. The bone marrow washing fluid was harvested and added in low-glucose Dulbecco's modified Eagle medium (L-DMEM) in combination with 10% fetal bovine serum (FBS; GIBCO, Grand Island, NY, USA) and 1% penicillin-streptomycin (Solarbio, Beijing, China). The single-cell suspension was collected and centrifuged for 5 min at 250 g with the supernatant abandoned. After resuspension in the fresh medium, cells were seeded in 6-well plates at 1 × 10^6^ cells/well. The medium was changed every 3 days. The morphology of cells at 3rd passage (Passage 3) was observed under a microscope. The differentiation abilities of osteogenesis, adipogenesis, and chondrogenesis were assessed using Alizarin Red (G1450, Solarbio), Oil Red O (G1262, Solarbio), and Alcian Blue (G2542, Solarbio), respectively. The expressions of BMSC surface markers CD29 (1 : 1,000, ab30394, Abcam, Cambridge, MA, USA), CD90 (1 : 1,000, ab25672), CD34 (1 : 1,000, ab81289), and CD45 (1 : 1,000, ab10558) were determined by flow cytometry.

### 2.4. Isolation of BMSC-EVs

Upon 80% confluence, BMSCs (Passages 3-6) were rinsed 3 times with phosphate-buffered saline (PBS). The medium was changed to a serum-free medium (Umibio, Shanghai, China) for 48 h culture, and the supernatant was then centrifuged for 10 min at 4°C and 300 g for the removal of cells and cell debris, for 10 min at 2,000 g for the removal of dead cells and larger EVs, for 30 min at 10,000 g for the removal of cell debris, and for 70 min at 100,000 g for the removal of the supernatant. The obtained precipitation was rinsed with PBS, resuspended, and centrifuged for 70 min at 100,000 g to discard the supernatant. EV precipitation was resuspended in 100 *μ*L PBS, and EV protein quantification was carried out by bicinchoninic acid (BCA) protein assay kits (P0012, Beyotime, Shanghai, China). A transmission electron microscope (JEOL, Tokyo, Japan) was used to observe EV characteristics, and the qNano system (Izon Science, Christchurch, New Zealand) was used for analysis [23]. The expression levels of EV surface markers CD63, CD81, TSG101, and Calnexin were measured by Western blot. In addition, BMSCs treated with 10 *μ*M GW4869 (Sigma-Aldrich, Merck KGaA, Darmstadt, Germany) were cultured with the supernatant as the negative control (GW).

Small interfering RNA of NORAD (si-NORAD, 5′-GCGGTTGGTCTTCATTCTA-3′) or its negative control si-NC (5′-TTCTCCGAACGTGTCACGT-3′) was delivered into BMSCs under the instructions of Lipofectamine 3000 (Invitrogen, Carlsbad, CA, USA). EVs isolated from the BMSCs were named EVs-si-NC and EVs-si-NORAD.

### 2.5. Cell Culture and Grouping

Human osteoblasts hFOB 1.19 and OS cell lines 143B, MG-63, Saos2, HOS, and U-2OS were procured from ATCC (Manassas, VA, USA). Saos2 and U-2OS cells were cultured in DMEM containing 10% FBS (GIBCO) and 1% penicillin-streptomycin with 5% CO_2_ at 37°C in a thermostat. Cells were assigned to 9 groups: the blank group, GW group, EV (25, 50, and 100 *μ*g/mL) group, EVs-si-NC group, EVs-si-NORAD group, EVs-si-NORAD + inhibitor NC group, EVs-si-NORAD + miR-877-3p inhibitor group, EVs-si-NORAD + pc-NC group, and EVs-si-NORAD + pc-CREBBP group. Cells in the blank and GW groups were cultured with PBS- and GW4869-treated BMSC supernatant. Cells in the EVs-si-NC and EVs-si-NORAD groups were, respectively, transfected with 100 *μ*g/mL EVs-si-NC and EVs-si-NORAD. Cells in the EVs-si-NORAD + inhibitor NC group, EVs-si-NORAD + miR-877-3p inhibitor group, EVs-si-NORAD + pc-NC group, and EVs-si-NORAD + pc-CREBBP group were transfected with 100 *μ*g/mL EVs-si-NORAD after separate transfection with inhibitor NC/miR-877-3p inhibitor or pc-NC/pc-CREBBP (GenePharma, Shanghai, China). Cell transfection was conducted using Lipofectamine 3000 (Invitrogen). Subsequent experiments were followed after 48 h transfection.

### 2.6. Immunofluorescence

EVs were labeled with PKH26 as instructed by the Red Fluorescent Cell Linker Mini kits (MINI26, Sigma-Aldrich) and centrifuged for 70 min at 4°C and 100,000 g for the removal of unbound dyes. After PBS rinsing, the labeled EVs were recentrifuged and resuspended in 100 *μ*L PBS. Once the confluence reached 80%, the medium of Saos2 and U-2OS cells was replaced, added with PKH26-labeled EVs, and incubated for 12 h. Following incubation, cells were rinsed with PBS, fixed in 4% paraformaldehyde (PFA), and stained with 4′,6-diamidino-2-phenylindole. The internalization of EVs by OS cells was observed under a laser confocal microscope (LSM5, Zeiss, Jena, Germany).

### 2.7. Cell Counting Kit-8 (CCK-8) Assay

Cells in different groups were seeded in 96-well plates at 2 × 10^4^ cells/well and added with 100 *μ*g/mL EVs at different treatments for 0, 24, 48, and 72 h. OS cell proliferation was detected by the CCK-8 kit (Beyotime). Absorbance at 450 nm was detected using the Elx800 Reader (Bio-Tek Instruments Inc., Winooski, VT, USA).

### 2.8. Transwell Assays

The invasion and migration of OS cells were detected using a Transwell chamber. Cell invasion evaluation was conducted using a Transwell chamber precoated with Matrigel (BD Bioscience, Franklin Lakes, NJ, USA), and the Transwell chamber for cell migration evaluation was Matrigel-free. After 24 h starvation in a serum-free medium prior to the experiment, cells were resuspended in the culture medium supplemented with 1% FBS. The apical chamber was seeded with 1 × 10^5^ cells, and the basolateral chamber was added with 600 *μ*L complete medium supplemented with 10% FBS. Following 24 h of incubation, the cells that remained in the apical chamber were removed using cotton swabs. Cells that passed through were fixed in 4% PFA and stained with hematoxylin. Five random visual fields were selected for observation and counting cells in each visual field.

### 2.9. Tube Formation Assay

The tube formation ability of OS cells was detected as per the provided instructions of Angiokit™ (TCS cell works, London, UK). In brief, 1 × 10^4^ cells were seeded in Matrigel and incubated for 6 h. Images were collected under a microscope. Cell-formed tubes were counted using the Image-Pro Plus 6.0 software (Media Cybernetics, Rockville, MD, USA). The average number of five visual fields was calculated.

### 2.10. Reverse Transcription-Quantitative Polymerase Chain Reaction (RT-qPCR)

The total RNA in cells and tumor tissues was extracted using a TRIzol kit (Invitrogen) as per the instructions and subsequently reverse transcribed using the PrimeScript RT Kit (TAKARA, Dalian, China), followed by qPCR using SYBR Premix Ex Taq II (TAKARA) on the 7500 Real-Time PCR system (ABI, Foster City, CA, USA). The expressions of NORAD and CREBBP were normalized to *β*-actin level, and the expression of miR-877-3p was normalized to U6 level. All results were computed using the 2^-*ΔΔ*CT^ method. The primer sequences were listed as follows: NORAD, 5′-GCCATTGGGCGAGACCTACCT-3′ (forward) and 5′-GTTCGGGACTTCGCTCACCTT-3′ (reverse); miR-877-3p, 5′-TCCTCTTCTCCCTCCTCCC-3′ (forward) and 5′-CTCTACAGCTATATTGCCAGCC-3′ (reverse); CREBBP, 5′-CGGCTCTAGTATCAACCCAGG-3′ (forward) and 5′-TTTTGTGCTTGCGGATTCAGT-3′ (reverse); *β*-actin, 5′-CATCCGTAAAGACCTCTATGCCAAC-3′ (forward) and 5′-ATGGAGCCACCGATCCACA-3′ (reverse); and U6, 5′-CTCGCTTCGGCAGCAC-3′ (forward) and 5′-ACGCTTCACGAATTTGC-3′ (reverse).

### 2.11. Western Blot

The total protein was extracted from cells and tissues using the radioimmunoprecipitation assay (RIPA) lysis buffer containing a protease inhibitor. Protein concentration was detected using the BCA protein quantitative kit (Beyotime). Then, a 30 *μ*g protein sample was separated by 10% sodium dodecyl sulfate-polyacrylamide gel electrophoresis and transferred onto the polyvinylidene fluoride membranes (Millipore, Bedford, MA, USA). The membranes were blocked for 2 h in 5% skim milk, followed by overnight incubation at 4°C with primary antibodies against CD63 (1 : 1,000, ab217345, Abcam, Cambridge, MA, USA), CD81 (1 : 1,000, ab109201, Abcam), TSG101 (1 : 1,000, ab30871, Abcam), Calnexin (1 : 1,000, ab241154, Abcam), CREBBP (1 : 1,000, ab2832, Abcam), and *β*-actin (1 : 5,000, ab6276, Abcam). After rinsing with Tris-buffered saline–Tween-20, the membranes were added with HRP-labeled goat anti-rabbit secondary antibody IgG (ab205718, 1 : 2,000, Abcam) at room temperature for 1 h. The enhanced chemiluminescence working solution was used for development. The gray value of the protein bands in Western blot images was quantified using the ImageJ software (Media Cybernetics) with *β*-actin as an internal control.

### 2.12. Dual-Luciferase Reporter Assay

The presumed binding site of miR-877-3p in NORAD and CREBBP was predicted on the LncBase Predicted v.2 and TarBase v.8 online tools (http://carolina.imis.athena-innovation.gr/diana_tools/web/index.php). The NORAD-3′-UTR fragments containing miR-877-3p wild-type (NORAD-WT) and mutant (NORAD-MUT) binding sites were inserted into the pmirGLO luciferase vectors (E1330, Promega, Madison, WI, USA). Additionally, the CREBBP-3′-UTR fragments containing miR-877-3p wild-type (CREBBP-WT) and mutant (CREBBP-MUT) binding sites were inserted into the pmirGLO luciferase vectors. Lipofectamine 3000 was used for cotransfection of NORAD-WT/NORAD-MUT and CREBBP-WT/CREBBP-MUT with miR-877-3p mimic or mimic NC and cultured for 48 h. Next, cells were harvested for the dual-luciferase assay (Promega). The luciferase activity was measured using the SpectraMax L fluorometer (Molecular Devices, Sunnyvale, CA, USA).

### 2.13. RNA Immunoprecipitation (RIP) Assay

RIP assay was carried out with the EZ Magna RIP™ kit (EMD Millipore, Billerica, MA, USA). Cells were lysed in a complete RIPA buffer containing protease and RNase inhibitors. The cell extraction solution was coincubated with RIP buffer containing magnetic beads labeled with human anti-AGO2 antibody (EMD Millipore) or IgG antibody. Finally, combined RNA was purified by TRIzol, and expressions of miR-877-3p and NORAD were determined by RT-qPCR.

### 2.14. Xenograft Tumors in Nude Mice

Balb/c mice (4 weeks old) were randomized into 4 groups (*N* = 12): the NC group, EV group, EVs-si-NC group, and EVs-si-NORAD group. Subsequently, mice were injected with 5 × 10^6^ Saos2 cells in the posterior scapula, followed by intravenous administration of 20 *μ*g EVs *via* the caudal vein two times a week for a week [24]. The tumor volume was calculated on the 7th, 14th, 21st, and 28th days using the formula [*V* = 0.5 × *L* (length) × *W* (width)^2^]. The nude mice were euthanized 4 weeks later *via* intraperitoneal administration of an overdose of pentobarbital sodium (800 mg/kg). Tumor tissues were subsequently removed for weight measurement and further analysis.

### 2.15. Immunohistochemistry

The prepared tumor tissue sections were incubated with CREBBP antibody (1 : 1,000, ab2832) and stained with HRP-labeled anti-rabbit IgG or diaminobenzidine. Moreover, the sections were incubated with a mouse secondary antibody and stained with EnVision G2 System/AP Rabbit Mouse Permanent Red (Dako, Glostrup, Denmark). Finally, the sections were counterstained with hematoxylin and observed under a fluorescence inverted microscope (Hitachi Limited, Tokyo, Japan).

### 2.16. Statistical Analysis

All results were presented in the form of mean ± standard deviation (SD). Data were analyzed and plotted using the GraphPad Prism 8 software. The independent-sample *t* test was adopted for pairwise comparisons while one-way analysis of variance (ANOVA) was adopted for multigroup comparisons. The post hoc test was conducted using Tukey's multiple comparisons test. The value of *p* < 0.05 was suggestive of statistical significance.

## 3. Results

### 3.1. Isolation and Identification of BMSCs and BMSC-EVs

**Figure 1:**
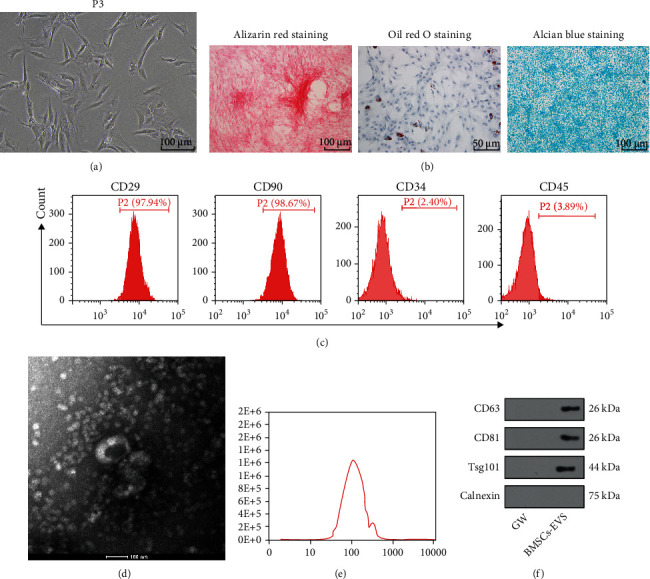
Isolation and identification of BMSCs and BMSC-EVs. (a) BMSC (P3) morphology observed under a microscope; (b) osteogenic, adipogenic, and chondrogenic differentiation of BMSCs detected by Alizarin Red, Oil Red O, and Alcian Blue staining; (c) expressions of BMSC surface markers CD29, CD90, CD34, and CD45 measured by flow cytometry; (d) BMSC-EV morphology observed under a TEM; (e) size distribution of BMSC-EVs measured by the qNano system; (f) expressions of BMSC-EV-specific markers CD63, CD81, TSG101, and Calnexin measured by Western blot. The BMSC culture supernatant after GW4869 intervention served as the negative control (GW).

As previously mentioned, MSCs can promote OS growth and metastasis [7]. However, whether EVs derived from BMSCs could affect OS progression remains to be investigated. The BMSCs isolated from the femur and tibia of mice reached 80% confluence at the 3rd passage (P3) and exhibited nest-like growth (Figure 1(a)). Alizarin Red, Oil Red O, and Alcian Blue staining demonstrated calcium deposition and lipid and acid mucopolysaccharide accumulation in BMSCs (Figure 1(b)). Moreover, the results of flow cytometry demonstrated positive expressions of CD29 and CD90 (≥90%) and negative expressions of CD34 and CD45 (≤2%) (Figure 1(c)). These results were suggestive of successful isolation of BMSCs. Subsequently, EVs were isolated from BMSCs *via* ultracentrifugation and exhibited an oval shape (Figure 1(d)), a diameter of 100 nm, and a concentration of 1.5 × 10^6^ particles/mL (Figure 1(e)). Western blot showed positive expressions of CD63, CD81, and TSG101 and negative expression of Calnexin, while no expressions of EV markers were detected in BMSCs supernatant interfered by GW4869 (Figure 1(f)). The above results stated the successful isolation of BMSC-EVs.

### 3.2. BMSC-EVs Facilitated OS Cell Proliferation, Invasion, Migration, and Angiogenesis

**Figure 2:**
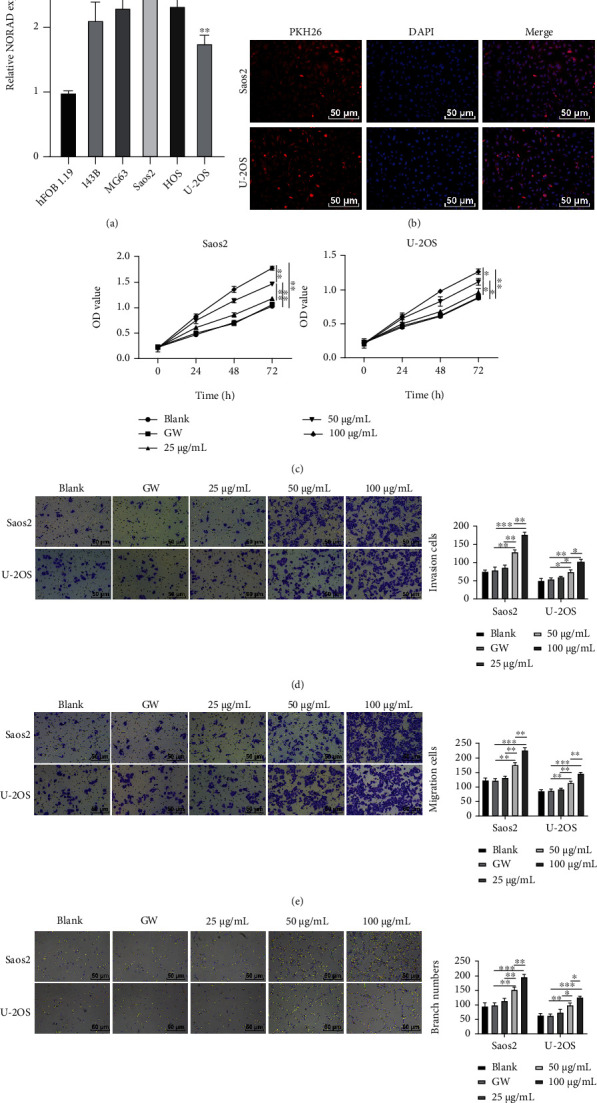
BMSC-EVs facilitated OS cell proliferation, invasion, migration, and angiogenesis. (a) Expression of NORAD in hFOB 1.19, 143B, MG63, Saos2, HOS, and U-2OS cells detected by RT-qPCR; (b) internalization of PKH-labeled EVs by OS cells detected by immunofluorescence; (c) effect of EVs on OS cell proliferation detected by CCK-8; (d) effect of EVs on OS cell invasion detected by the Transwell assay; (e) effect of EVs on OS cell migration detected by the Transwell assay; (f) effect of EVs on OS cell tube formation detected by tube formation assay. Cell experiment was repeated 3 times. Data were presented in the form of mean ± SD. One-way ANOVA was used for comparisons among multigroups, and Tukey's multiple comparisons test was applied for a post hoc test. ^∗^*p* < 0.05, ^∗∗^*p* < 0.01, ^∗∗∗^*p* < 0.001.

NORAD has been documented to be upregulated in OS [18]. RT-qPCR was conducted to further clarify the expression of NORAD in OS cells. The results indicated an upregulation of NORAD in OS cells, among which Saos2 cells showed the highest expression while U-2OS cells showed the lowest (Figure 2(a)). Thereafter, Saos2 and U-2OS cells were used for subsequent experiments. Then, Saos2 and U-2OS cells cocultured with EVs showed red fluorescence, indicating that EVs could be internalized by OS cells (Figure 2(b)). Subsequently, OS cells' proliferative ability was detected at different time points after treatment with different concentrations of EVs. Compared to that in the GW group, a stronger proliferation of Saos2 and U-2OS cells was observed in the EV group, and the proliferation enhanced with the increase of EV concentration (Figure 2(c)). Similarly, the invasion and migration of Saos2 and U-2OS cells and angiogenesis were increased with the increase of EV concentration (Figures 2(d)–2(f)).

### 3.3. BMSC-EVs Promoted OS Cell Proliferation, Invasion, Migration, and Angiogenesis by Carrying NORAD

**Figure 3:**
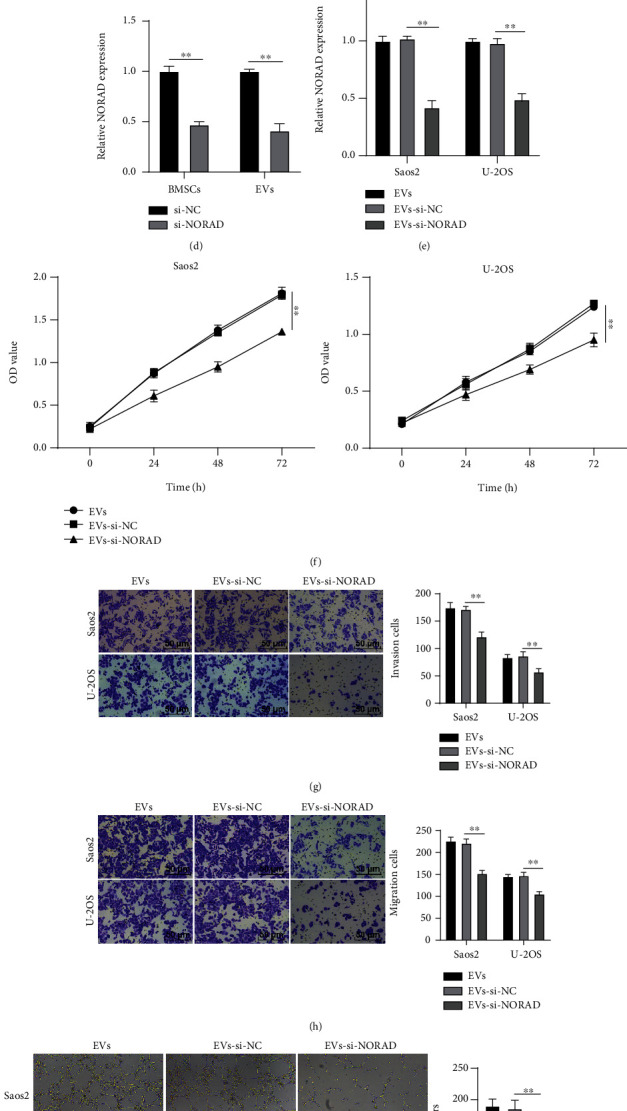
BMSC-EVs promoted OS cell proliferation, invasion, migration, and angiogenesis by carrying NORAD. (a) Effect of EVs on NORAD expression in OS cells detected by RT-qPCR; (b) expression of NORAD in EVs detected by RT-qPCR; (c) expression of NORAD in EVs and OS cells detected by RT-qPCR; (d) expression of NORAD in BMSCs and EVs after silencing NORAD detected by RT-qPCR; (e) expression of NORAD in OS cells detected by RT-qPCR; (f) OS cell proliferation detected by CCK-8; (g) OS cell invasion detected by the Transwell assay; (h) OS cell migration detected by the Transwell assay; (i) OS cell tube formation detected by tube formation assay. Cell experiment was repeated 3 times. Data were presented in the form of mean ± SD. The independent-sample *t* test was used for pairwise comparisons, one-way ANOVA for comparisons among multigroups, and Tukey's multiple comparisons test was used for a post hoc test. ^∗^*p* < 0.05, ^∗∗^*p* < 0.01, ^∗∗∗^*p* < 0.001.

We subsequently investigated whether BMSC-EVs could facilitate proliferation, invasion, migration, and angiogenesis of OS cells by carrying NORAD. RT-qPCR measured NORAD expression after EV treatment and demonstrated an increase in NORAD expression in the 100 *μ*g/mL EV group (Figure 3(a)). Moreover, NORAD was highly expressed in EVs, and its expression was higher in EVs than that in OS cells (Figures 3(b) and 3(c)), which implied that EVs might carry NORAD into OS cells. si-NC and si-NORAD were introduced into BMSCs for further clarification. RT-qPCR exhibited decreased expression of NORAD in BMSCs transfected with si-NORAD and the extracted BMSC-EVs (Figure 3(d)). Subsequently, 100 *μ*g/mL EVs-si-NC and EVs-si-NORAD were separately introduced into Saos2 and U-2OS cells. Compared to the EVs-si-NC group, the EVs-si-NORAD group showed decreased expression of NORAD in OS cells (Figure 3(e)). Additionally, the EVs-si-NORAD group exhibited weakened abilities of proliferation (Figure 3(f)), invasion (Figure 3(g)), migration (Figure 3(h)), and angiogenesis (Figure 3(i)) in Saos2 and U-2OS cells relative to the EVs-si-NC group. The above results elicited that silencing NORAD abolished the promotional function of EVs on malignant behaviors of OS cells and ascertained that BMSC-EVs accelerated OS cell proliferation, invasion, migration, and angiogenesis by carrying NORAD.

### 3.4. NORAD Carried by BMSC-EVs Upregulated CREBBP by Sponging miR-877-3p

**Figure 4:**
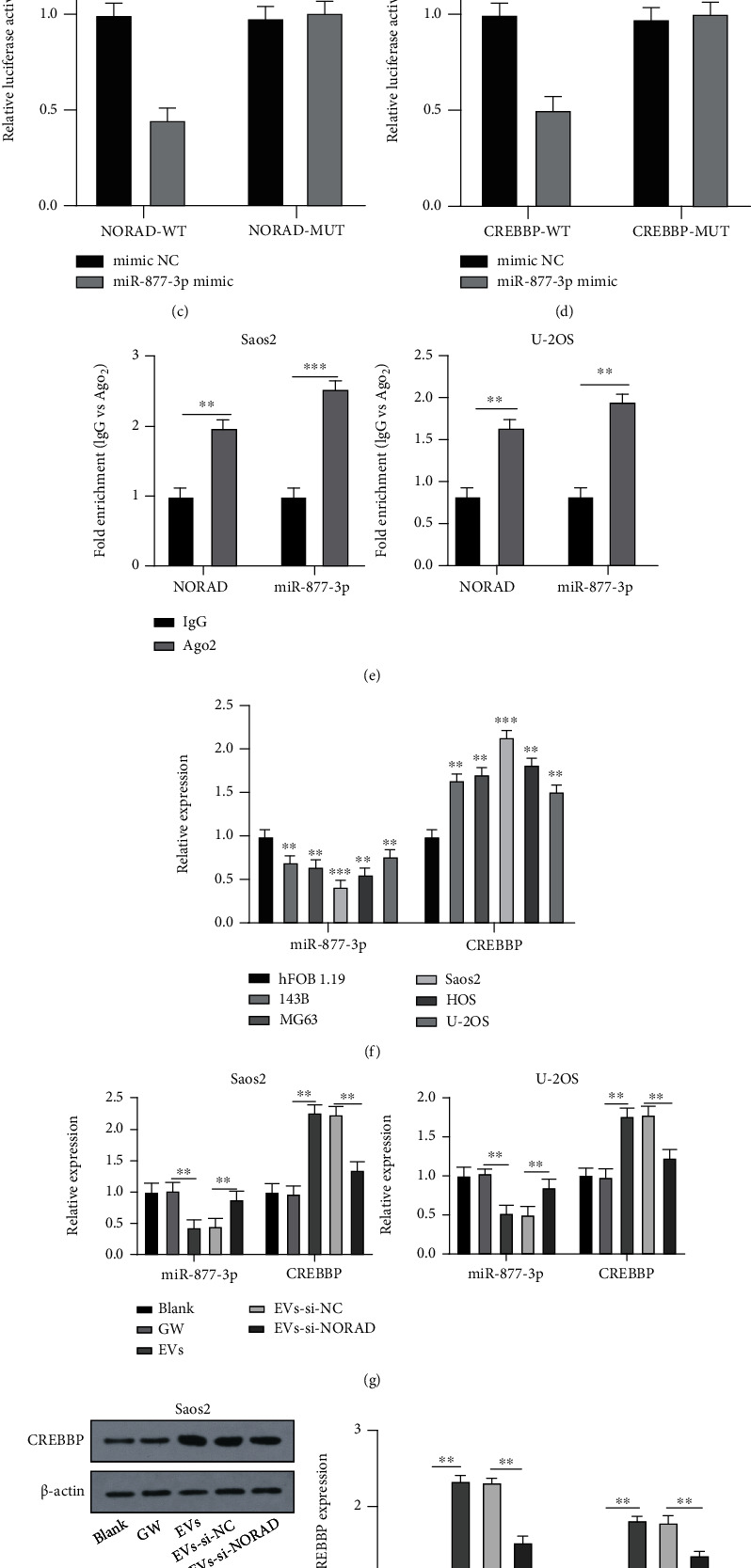
NORAD carried by BMSC-EVs upregulated CREBBP by sponging miR-877-3p. (a) Potential binding site of NORAD and miR-877-3p predicted on the LncBase Predicted v.2; (b) potential binding site of miR-877-3p and CREBBP predicted on the TarBase v.8; (c) binding relationship of NORAD and miR-877-3p validated by the dual-luciferase assay; (d) binding relationship of miR-877-3p and CREBBP validated by the dual-luciferase assay; (e) binding relationship of NORAD and miR-877-3p verified by the RIP assay; (f) expressions of miR-877-3p and CREBBP in hFOB 1.19, 143B, MG63, Saos2, HOS, and U-2OS cells detected by RT-qPCR; (g) expressions of miR-877-3p and CREBBP in OS cells detected by RT-qPCR; (h, i) expression of CREBBP in OS cells detected by Western blot. Cell experiment was repeated 3 times. Data were presented in the form of mean ± SD. The independent-sample *t* test was used for pairwise comparisons, one-way ANOVA for comparisons among multigroups, and Tukey's multiple comparisons test was used for a post hoc test. ^∗∗^*p* < 0.01, ^∗∗∗^*p* < 0.001.

To expound the specific regulatory mechanism of NORAD in EV-mediated OS process, LncBase Predicted v.2 was utilized to predict the downstream miRNAs of NORAD (Supplementary Figure S1). There is evidence that miR-877-3p is engaged in the modulation of OS progression and is able to suppress OS formation and angiogenesis when overexpressed [21, 25]. TarBase v.8 was used to predict the downstream mRNAs of miR-877-3p, and CREBBP was obtained (Supplementary Figure S2). The potential binding sites among NORAD, CREBBP, and miR-877-3p are illustrated in Figures 4(a) and 4(b). A previous study has indicated the high expression of CREBBP in OS [22], implying its involvement in OS progression. Hence, we speculated that EVs might modulate the OS process *via* the NORAD/miR-877-3p/CREBBP axis. The binding relationships were validated by the dual-luciferase reporter assay. Cotransfection of miR-877-3p mimic and NORAD-WT (CREBBP-WT) reduced the relative luciferase activity while cotransfection of miR-877-3p mimic and NORAD-MUT (CREBBP-MUT) had no significant effect on the relative luciferase activity (Figures 4(c) and 4(d)). Additionally, the RIP assay further verified the binding relationship between NORAD and miR-877-3p (Figure 4(e)). Subsequent RT-qPCR exhibited decreased expression of miR-877-3p and increased expression of CREBBP in OS cells, as well as the lowest expression of miR-877-3p in Saos2 cells and highest expression of miR-877-3p in U-2OS cells, while the opposite trend was observed in the expression patterns of CREBBP (Figure 4(f)). We subsequently investigated the regulatory function of NORAD on the miR-877-3p/CREBBP in OS cells. RT-qPCR showed diminished expression of miR-877-3p and augmented expression of CREBBP in Saos2 and U-2OS cells in the EVs group, which was partially inverted by silencing NORAD (Figure 4(g)). The expression patterns of CREBBP were verified in Western blot (Figures 4(h) and 4(i)). These results together indicated that NORAD carried by BMSC-EVs enhanced the expression of CREBBP by sponging miR-877-3p.

### 3.5. Downregulation of miR-877-3p Abolished the Inhibitory Function of EVs by Silencing NORAD on OS Cell Invasion, Migration, and Angiogenesis

**Figure 5:**
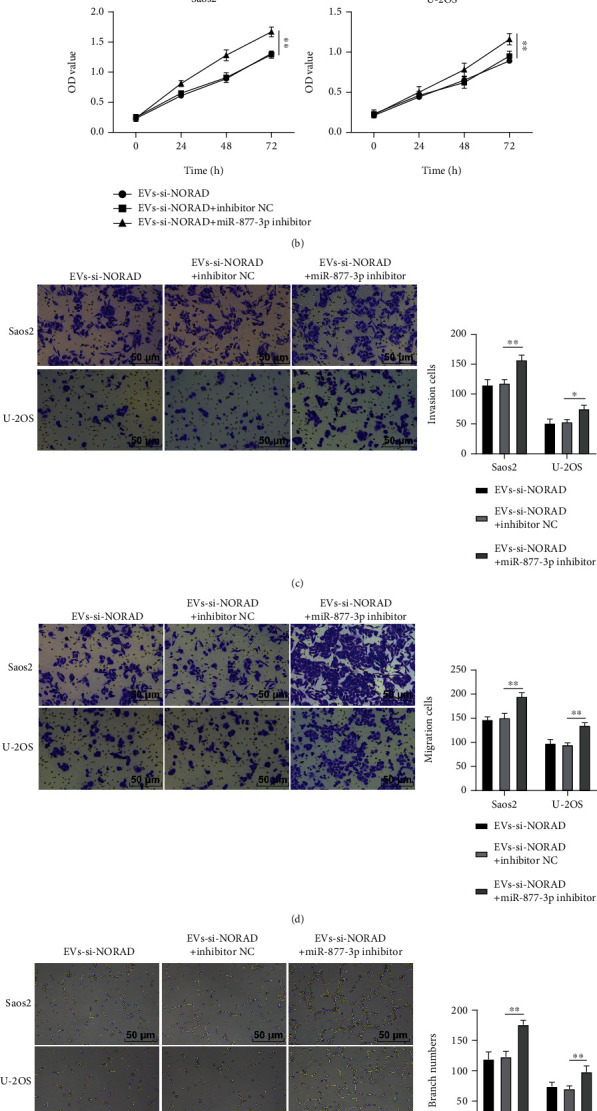
Downregulation of miR-877-3p abolished the inhibitory function of silencing NORAD on OS cell invasion, migration, and angiogenesis. (a) Expressions of miR-877-3p and CREBBP in OS cells detected by RT-qPCR; (b) OS cell proliferation detected by CCK-8; (c) OS cell invasion detected by the Transwell assay; (d) OS cell migration detected by the Transwell assay; (e) OS cell tuber formation detected by tube formation assay. Cell experiment was repeated 3 times. Data were presented in the form of mean ± SD. One-way ANOVA was used for comparisons among multigroups, and Tukey's multiple comparisons test was applied for a post hoc test. ^∗^*p* < 0.05, ^∗∗^*p* < 0.01.

We performed a combined experiment to further confirm the involvement of miR-877-3p in the regulation of NORAD on OS cells. Relative to cells in the EVs-si-NORAD group, Saos2 and U-2OS cells in the EVs-si-NORAD + miR-877-3p inhibitor group showed diminished expression of miR-877-3p and augmented expression of CREBBP (Figure 5(a)). In addition, the proliferation (Figure 5(b)), invasion (Figure 5(c)), migration (Figure 5(d)), and angiogenesis (Figure 5(e)) of Saos2 and U-2OS cells in the EVs-si-NORAD + miR-877-3p inhibitor group were significantly promoted relative to those in the EVs-si-NORAD group. In summary, downregulation of miR-877-3p could avert the inhibitory effect of EVs by silencing NORAD on OS cell invasion, migration, and angiogenesis.

### 3.6. Overexpression of CREBBP Annulled the Inhibitory Function of EVs by Silencing NORAD on OS Cell Invasion, Migration, and Angiogenesis

**Figure 6:**
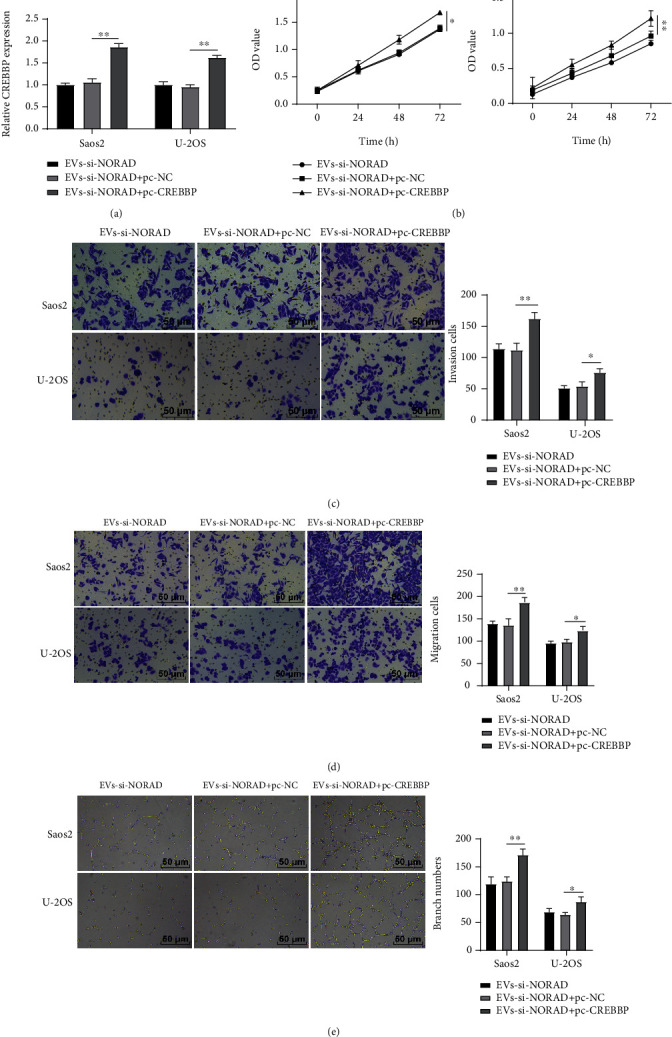
Overexpression of CREBBP annulled the inhibitory function of EVs by silencing NORAD on OS cell invasion, migration, and angiogenesis. (a) Expression of CREBBP in OS cells detected by RT-qPCR; (b) OS cell proliferation detected by CCK-8; (c) OS cell invasion detected by the Transwell assay; (d) OS cell migration detected by the Transwell assay; (e) OS cell tube formation detected by tube formation assay. Cell experiment was repeated 3 times. Data were presented in the form of mean ± SD. One-way ANOVA was used for comparisons among multigroups, and Tukey's multiple comparisons test was applied for a post hoc test. ^∗^*p* < 0.05, ^∗∗^*p* < 0.01.

To validate the participation of CREBBP in the regulation of NORAD on the biological behaviors of OS cells, the eukaryotic expression vector of CREBBP was constructed to alter its expression. Saos2 and U-2OS cells in the EVs-si-NORAD group exhibited an upregulation of CREBBP after transfection with pc-CREBBP (Figure 6(a)). Overexpression of CREBBP facilitated Saos2 and U-2OS cell proliferation (Figure 6(b)), invasion (Figure 6(c)), migration (Figure 6(d)), and angiogenesis (Figure 6(e)) and reversed the inhibitory effect of EVs by silencing NORAD.

### 3.7. BMSC-EVs Promoted Tumor Growth *via* the miR-877-3p/CREBBP Axis by Carrying NORAD

**Figure 7:**
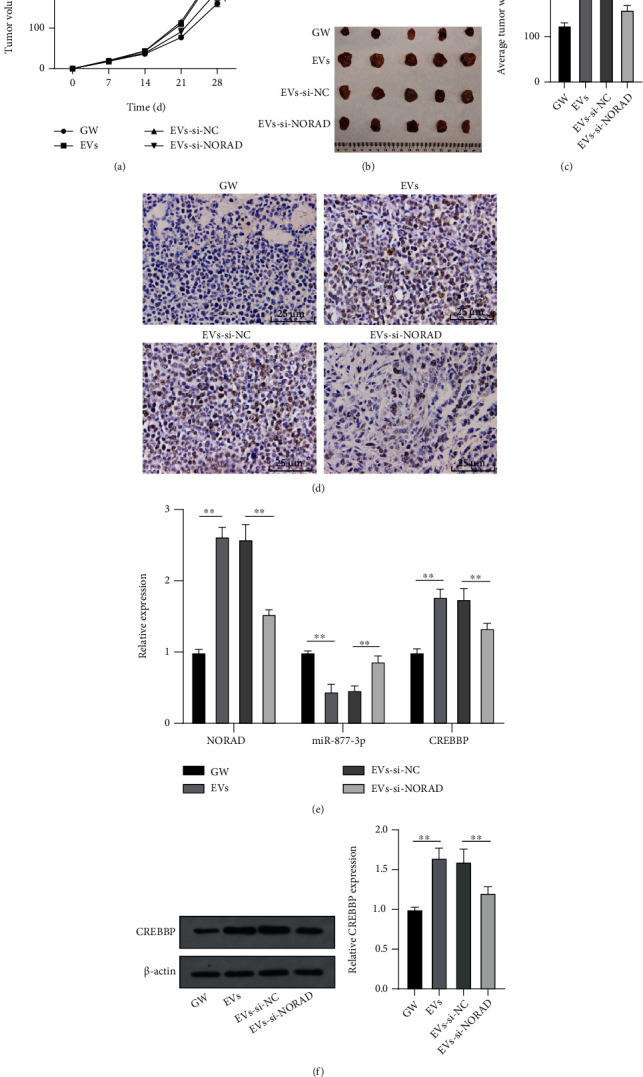
BMSC-EVs promoted tumor growth *via* the miR-877-3p/CREBBP axis by carrying NORAD. (a) Tumor volume of nude mice; (b) images of tumor; (c) tumor weight; (d) positive expression of Ki-67 in tumors of nude mice detected by immunohistochemistry; (e) expressions of NORAD, miR-877-3p, and CREBBP in tumor tissues of nude mice detected by RT-qPCR; (f) expression of CREBBP in tumor tissues of nude mice detected by Western blot. *N* = 6. ^∗∗^*p* < 0.01.

Finally, Saos2 cells were subcutaneously injected into the scapula of nude mice to further verify the action of BMSC-EVs on tumor growth. Compared to the GW group, the EV group exhibited increased tumor growth speed and weight, while the EVs-si-NORAD group exhibited significantly decreased tumor growth speed and weight (Figures 7(a)–7(c)). Compared to the EV group, diminished Ki-67-positive expression was shown in the EVs-si-NORAD group (Figure 7(d)). Furthermore, expressions of NORAD and CREBBP were augmented while miR-877-3p was diminished in tumor tissues of the EV group, and EVs by silencing NORAD could antagonize the trend (Figures 7(e) and 7(f)). Collectively, the *in vivo* experiment validated that BMSC-EVs facilitated OS tumorigenesis *via* the miR-877-3p/CREBBP axis by carrying NORAD.

## 4. Discussion

OS is a primary bone tumor commonly diagnosed among children and adolescents with the characteristic of early metastasis [26, 27]. LncRNAs have long been considered potential targets for the advanced management of OS [28]. LncRNA NORAD has been documented to facilitate OS progression by targeting its downstream miRNA [18]. In the present study, we elicited that BMSC-EV-derived NORAD promoted OS cell invasion, migration, and angiogenesis *via* miR-877-3p-mediated CREBBP.

BMSC-EVs are capable of promoting OS cell proliferation by carrying lncRNAs [29]. To further ascertain the results, we successfully isolated and identified BMSCs and BMSC-EVs. Recently, lncRNA NORAD was found to be upregulated in OS cell lines and clinical samples [30]. Therefore, we subsequently detected the expression of NORAD in normal osteoblasts and OS cell lines and then observed an increased expression of NORAD in OS cell lines, among which Saos2 cells showed the highest expression while U-2OS cells showed the lowest. Saos2 and U-2OS cells were used for subsequent experiments due to their differential expression patterns. The immunofluorescence results demonstrated fluorescence in Saos2 and U-2OS cells cocultured with EVs, suggestive of internalization of EVs by OS cells. The subsequent CCK-8, Transwell, and tube formation assays revealed enhanced proliferative, invasive, migratory, and angiogenic abilities in Saos2 and U-2OS cells after EV treatment in a concentration-dependent manner. BMSC-EVs have been documented to facilitate OS cell proliferation, migration, and invasion [31]. We herein ascertained that BMSC-EVs exerted a promotional effect on OS cell proliferation, invasion, migration, and angiogenesis. To validate whether BMSC-EVs could carry lncRNA NORAD into OS cells, we detected the NORAD expression in BMSCs and BMSC-EVs and subsequently noticed a significantly increased expression of NORAD in EVs relative to OS cells, indicative of the ability of EVs to carry NORAD into OS cells. We then investigated the role of BMSC-EV-derived NORAD in OS cells by silencing NORAD in BMSCs and BMSC-EVs and subsequently treating Saos2 and U-2OS cells with EVs-si-NORAD. The results elicited a decreased expression of NORAD in Saos2 and U-2OS cells, along with compromised abilities of proliferation, invasion, migration, and angiogenesis after treating with EVs-si-NORAD. After lncRNA NORAD was overexpressed in OS cell lines Saos-2 and 143B *via* transfection with NORAD siRNA, the cell proliferative and invasive abilities and *in vivo* tumor growth were all markedly suppressed [30], which was tallied with our finding that BMSC-EVs carried NORAD into OS cells to accelerate OS cell growth.

The possible mechanism of BMSC-EVs in OS involving NORAD was subsequently explored. miRNA and mRNA regulation is implicated in the modulation of NORAD in OS [32]. Thus, we predicted the downstream miRNAs and mRNAs of NORAD, among which we identified miR-877-3p and CREBBP that presented an association with OS [21, 22]. Thereafter, we predicted the binding sites among NORAD, miR-877-3p, and CREBBP and further validated their binding relationship. RT-qPCR exhibited an increased expression of CREBBP and a decreased expression of miR-877-3p in OS cells. miR-877-3p was at the highest level in Saos2 cells and the lowest level in U-2OS cells while the opposite trend was noticed regarding the expression of CREBBP. After silencing NORAD, miR-877-3p was upregulated and CREBBP was downregulated in OS cells treated with EVs. The binding relationship between CREBBP and NORAD has been identified earlier [33]. Similarly, the current study ascertained that NORAD carried by BMSC-EVs promoted CREBBP expression by sponging miR-877-3p.

To further confirm the action of miR-877-3p in NORAD-mediated manipulation on OS cells, we performed a joint experiment to downregulate miR-877-3p in OS cells with EVs-si-NORAD. The enhanced proliferative, invasive, migratory, and angiogenic abilities were subsequently observed in OS cells transfected with the EVs-si-NORAD + miR-877-3p inhibitor. miR-877-3p could inhibit OS cells' proliferative, invasive, and migratory abilities *via* targeting gamma-glutamylcyclotransferase [34]. Altogether, downregulation of miR-877-3p partly annulled the inhibitory effect of EVs by silencing NORAD on OS cell invasion, migration, and angiogenesis. Meanwhile, to validate the involvement of CREBBP in NORAD-mediated regulation on OS cell biological behaviors, CREBBP was upregulated in Saos2 and U-2OS cells using pc-CREBBP transfection. Our results illustrated that CREBBP overexpression facilitated the proliferative, invasive, migratory, and angiogenic abilities of Saos2 and U-2OS cells, suggestive of the abolishment of EVs-si-NORAD. CREBBP stimulates tumor cell growth in hepatocellular carcinoma [35] and positively modulates angiogenesis [36]. There is little study on the regulation of CREBBP on OS cells. Likewise, our results initially elucidated that overexpression of CREBBP averted the inhibitory effect of EVs by silencing NORAD on OS cell invasion, migration, and angiogenesis.

In addition, the *in vivo* experiments showed increased tumor growth and weight in nude mice administered with EVs and decreased tumor growth and weight in nude mice administered with EVs-si-NORAD. On the other hand, expressions of NORAD and CREBBP were augmented while miR-877-3p was diminished in tumor tissues of nude mice administered with EVs, which was abrogated by administration of EVs by silencing NORAD. NORAD also contributes to tumor growth in colorectal cancer as a ceRNA of miR-202-5p [37]. miR-877-3p negatively manipulates the growth of gastric cancer [38]. miR-181d exerts antitumor effects in glioblastoma by targeting CREBBP [39]. Altogether, BMSC-EVs promoted tumor growth by carrying NORAD to regulate the miR-877-3p/CREBBP axis.

**Figure 8:**
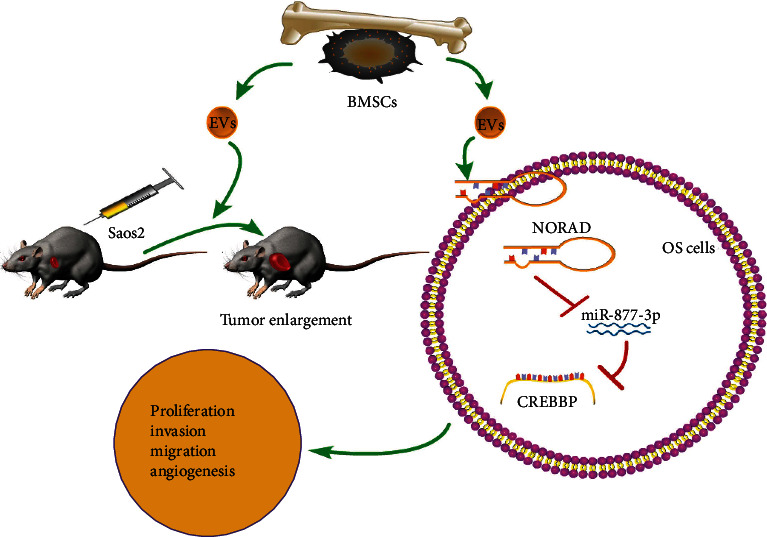
BMSC-EV-derived lncRNA NORAD promoted OS cell proliferation, invasion, migration, and angiogenesis and thus facilitated OS progression *via* miR-877-3p-mediated CREBBP.

In conclusion, BMSC-EV-derived lncRNA NORAD promoted OS cell proliferation, invasion, migration, and angiogenesis and thus facilitated OS progression *via* modulating CREBBP through miR-877-3p (Figure 8). The present study was flawed, though. Mechanically, acetyltransferase CREBBP only participated in OS progression *via* the ceRNA mechanism as the downstream target of miRNAs. The possibility of CREBBP affecting OS progression by mediating acetylation remains to be clarified. Hence, future studies should be implemented to further study whether acetylation modification occurs in the miRNA promoter region and mediates the expressions of downstream target genes to manipulate OS development.

## Data Availability

All the data generated or analyzed during this study are included in this published article.

## Supplementary Materials

Supplementary 1 Underlying binding site of NORAD and miR-877-3p predicted using the LncBase Predicted v.2 software.

Supplementary 2 Underlying binding sites of miR-877-3p and CREBBP predicted using the TarBase v.8 software.
