# Supplementary material for: BMSC-EV-derived lncRNA NORAD Facilitates Migration, Invasion, and Angiogenesis in Osteosarcoma Cells by Regulating CREBBP via Delivery of miR-877-3p
Source: Oxid Med Cell Longev. 2022 Mar 1;2022:8825784. doi: 10.1155/2022/8825784 (PMC8906129; doi:10.1155/2022/8825784)
Supplement: Supplementary 1 — Underlying binding site of NORAD and miR-877-3p predicted using the LncBase Predicted v.2 software. [file 8825784.f1.pdf]

miRNA

lncRNA

or Search by location

ENSG00000260032 ✕

GO to Experimental module ⇄

« 5 6 7 8 9 10 11 12 13 14 »

Gene	miRNA	Score	DIANA Links
LINC00657	hsa-miR-211-5p	0.795	mT TB lnE mP
LINC00657	hsa-let-7g-3p	0.795	mT TB lnE mP
LINC00657	hsa-miR-4678	0.795	mT TB lnE mP
LINC00657	hsa-miR-3679-3P	0.794	mT TB lnE mP
LINC00657	hsa-miR-4307	0.793	mT TB lnE mP
LINC00657	hsa-miR-877-3P	0.791	mT TB lnE mP
LINC00657	hsa-miR-4282	0.791	mT TB lnE mP
LINC00657	hsa-miR-4733-5P	0.791	mT TB lnE mP
LINC00657	hsa-miR-204-5P	0.791	mT TB lnE mP
LINC00657	hsa-let-7c-3p	0.789	mT TB lnE mP
LINC00657	hsa-miR-4662b	0.788	mT TB lnE mP
LINC00657	hsa-miR-577	0.788	mT TB lnE mP
