# Supplementary figures and images for: BMSC-EV-derived lncRNA NORAD Facilitates Migration, Invasion, and Angiogenesis in Osteosarcoma Cells by Regulating CREBBP via Delivery of miR-877-3p
Source: Oxid Med Cell Longev. 2022 Mar 1;2022:8825784. doi: 10.1155/2022/8825784 (PMC8906129; doi:10.1155/2022/8825784)

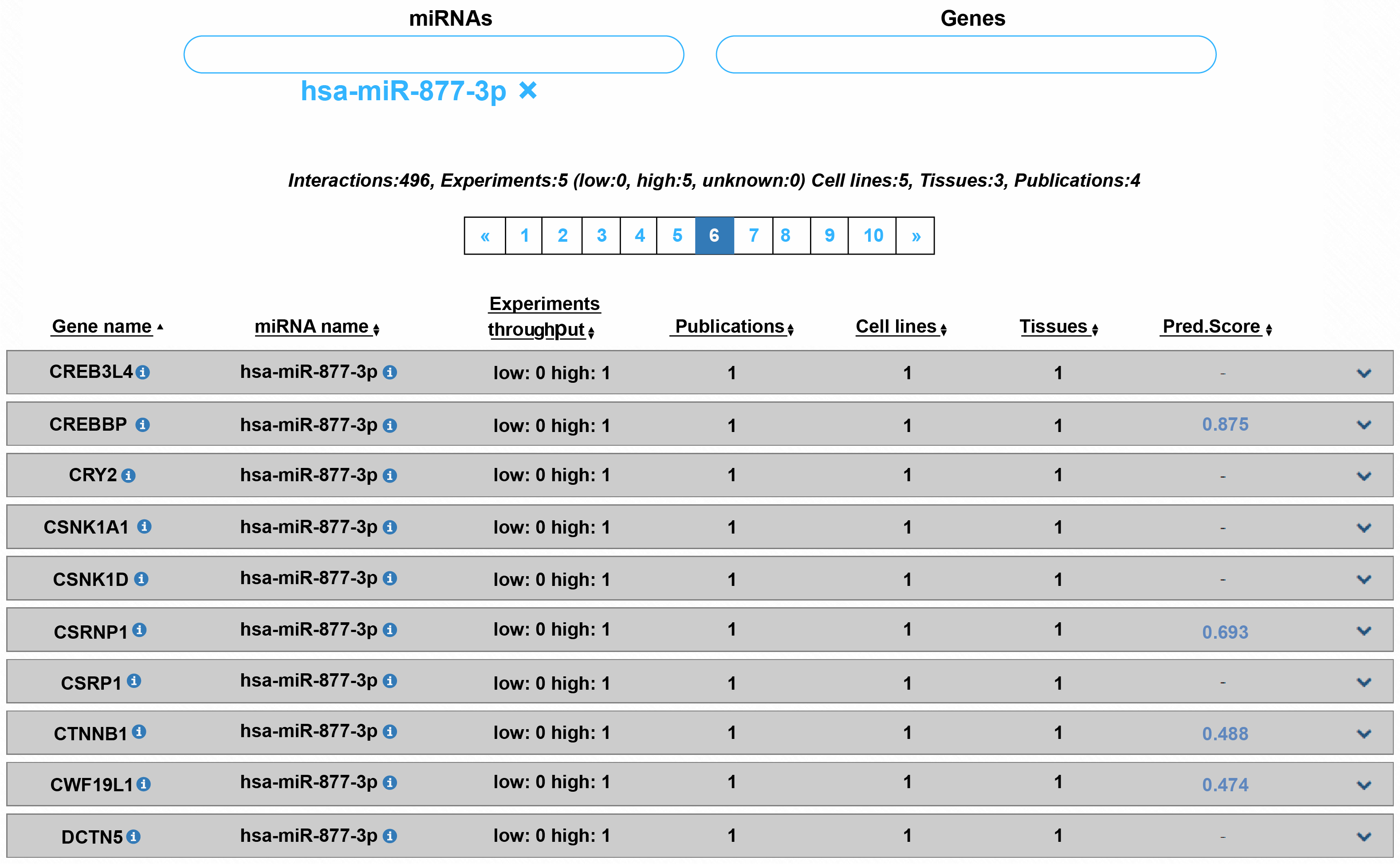

Supplement: Supplementary 2 — Underlying binding sites of miR-877-3p and CREBBP predicted using the TarBase v.8 software. [file 8825784.f2.tiff]
